# The TR-*ic*OS setup at the ESRF: time-resolved microsecond UV–Vis absorption spectroscopy on protein crystals

**DOI:** 10.1107/S2059798323010483

**Published:** 2024-01-01

**Authors:** Sylvain Engilberge, Nicolas Caramello, Sergei Bukhdruker, Martin Byrdin, Thierry Giraud, Philippe Jacquet, Damien Scortani, Rattana Biv, Hervé Gonzalez, Antonin Broquet, Peter van der Linden, Samuel L. Rose, David Flot, Taras Balandin, Valentin Gordeliy, J. Mia Lahey-Rudolph, Manfred Roessle, Daniele de Sanctis, Gordon A. Leonard, Christoph Mueller-Dieckmann, Antoine Royant

**Affiliations:** a European Synchrotron Radiation Facility, 71 Avenue des Martyrs, CS 40220, 38403 Grenoble CEDEX 9, France; b Université Grenoble Alpes, CNRS, CEA, Institut de Biologie Structurale (IBS), 71 Avenue des Martyrs, CS 10090, 38044 Grenoble CEDEX 9, France; c Hamburg Centre for Ultrafast Imaging, Universität Hamburg, HARBOR, Luruper Chaussee 149, 22761 Hamburg, Germany; d Institute of Biological Information Processing (IBI-7: Structural Biochemistry), Forschungszentrum Jülich, Jülich, Germany; e JuStruct: Jülich Center for Structural Biology, Forschungszentrum Jülich, Jülich, Germany; f PSCM (Partnership for Soft Condensed Matter), ESRF, 71 Avenue des Martyrs, 38000 Grenoble, France; g Technische Hochschule Lübeck – University of Applied Sciences, Mönkhofer Weg 239, 23562 Lübeck, Germany; STFC Rutherford Appleton Laboratory, United Kingdom

**Keywords:** pump–probe spectroscopy, photoactivable proteins, *in crystallo* optical spectroscopy, bacteriorhodopsin, serial synchrotron crystallography

## Abstract

The technique of time-resolved macromolecular crystallography (TR-MX) has recently been rejuvenated at synchrotrons, resulting in the design of dedicated beamlines. Using pump–probe schemes, this should make the mechanistic study of photoactive proteins and other suitable systems possible with time resolutions down to microseconds. In order to identify relevant time delays, time-resolved spectroscopic experiments directly performed on protein crystals are often desirable. To this end, an instrument has been built at the *ic*OS Lab (*in crystallo* Optical Spectroscopy Laboratory) at the European Synchrotron Radiation Facility using reflective focusing objectives with a tuneable nanosecond laser as a pump and a microsecond xenon flash lamp as a probe, called the TR-*ic*OS (time-resolved *ic*OS) setup. Using this instrument, pump–probe spectra can rapidly be recorded from single crystals with time delays ranging from a few microseconds to seconds and beyond. This can be repeated at various laser pulse energies to track the potential presence of artefacts arising from two-photon absorption, which amounts to a power titration of a photoreaction. This approach has been applied to monitor the rise and decay of the M state in the photocycle of crystallized bacteriorhodopsin and showed that the photocycle is increasingly altered with laser pulses of peak fluence greater than 100 mJ cm^−2^, providing experimental laser and delay parameters for a successful TR-MX experiment.

## Abstract 2

A setup to measure time-resolved UV–Vis absorption spectra from small protein crystals using a pump–probe scheme with microsecond-to-second delays is described.

## 1. Introduction

**Figure 1:**
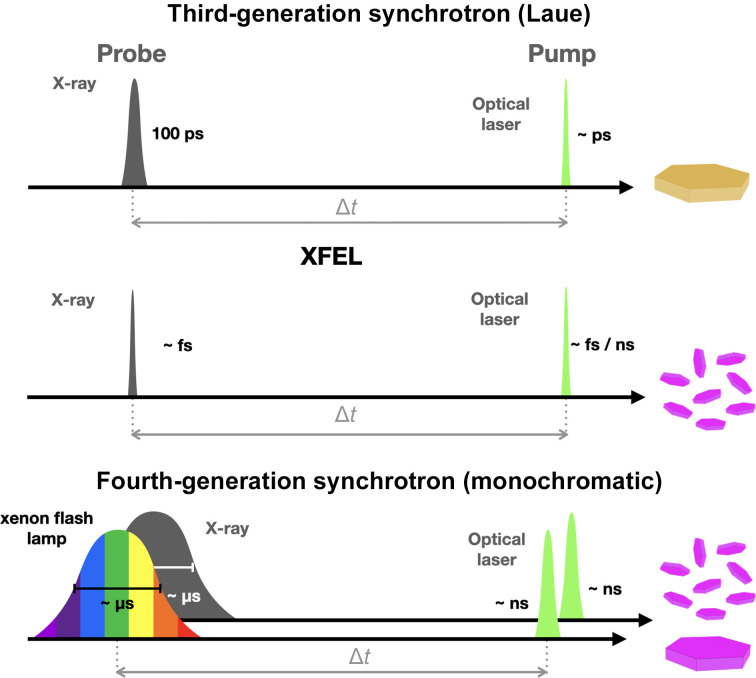
Schematics showing the principles of a time-resolved pump–probe experiment using picosecond Laue diffraction on single crystals at third-generation synchrotrons (top), SFX at XFELs on microcrystals (middle) and SSX at fourth-generation synchrotrons (bottom). The optical laser pump signal is shown in green, X-ray pulses in grey and white-light pulses in rainbow colours. The crystal colours correspond to the prototypal photoactive yellow protein (PYP) studied by Laue diffraction and to bacteriorhodopsin for XFEL and monochromatic synchrotron diffraction. The microsecond X-ray pulse in the lower panel is shown for simplicity with a Gaussian profile, when it should have a trapezoidal shape, since it is generated using a rotating chopper.

Time-resolved macromolecular crystallography (TR-MX) was successfully developed in the 1990s, taking advantage of the polychromatic, intense single X-ray pulses of third-generation synchrotron storage rings (Moffat, 2019 ▸). The experiments were performed using a pump–probe scheme (Fig. 1 ▸) with a picosecond optical laser as the pump. The maximum time resolution of these experiments eventually reached the ∼100 ps duration of a single X-ray bunch. Laue TR-MX produced remarkable molecular movies of the photolysis and rebinding reaction of carbon monoxide in complex with myoglobin (Schotte *et al.*, 2003 ▸) and of the photocycle of the photoactive yellow protein (PYP; Jung *et al.*, 2013 ▸). However, the technique remained limited to biological systems which could yield low-mosaicity crystals of significant size (hundreds of micrometres in at least two directions) and for which the photoreaction could be repeatedly cycled without compromising the diffraction quality of the crystals.

The development of hard X-ray free-electron lasers (XFELs) revived the field of TR-MX (Brändén & Neutze, 2021 ▸). The duration of the very intense X-ray pulses that they produce can be as low as a few femtoseconds (resulting in the technique being called TR-SFX, ‘time-resolved serial femtosecond crystallography), but in contrast to Laue TR-MX the limitation on the time resolution is the pulse duration of the pumping optical laser (∼100 fs; Fig. 1 ▸). The principle of recording one single diffraction image from a given sample at an XFEL before it is destroyed called for the use of a large number of micrometre-sized crystals (‘microcrystals’) and led to the development of serial crystallography (SX). It was soon realized that SX could be performed at third-generation synchrotrons, eventually leading to TR-SMX (serial millisecond crystallography; Nogly *et al.*, 2015 ▸) and TR-SSX (serial synchrotron X-ray crystallography; Diederichs & Wang, 2017 ▸). In particular, TR-SSX was applied to microbial rhodopsins, such as bacteriorhodopsin, to reach 5 ms time resolution (Weinert *et al.*, 2019 ▸) and, very recently, to a xenorhodopsin with 500 µs time resolution (Kovalev *et al.*, 2023 ▸).

Over the last decade, third-generation synchrotrons have planned the design and construction of significantly improved machine layouts leading to much higher brilliance and coherence of the X-ray beams produced. The European Synchrotron Radiation Facility (ESRF) in Grenoble, France and MAX IV in Lund, Sweden are the first two fourth-generation synchrotrons to have carried out the construction of specific TR-SSX beamlines, taking advantage of the much higher brilliance to produce large-bandwidth monochromatic microsecond pulses. ID29-SMX at the ESRF is the first such beamline (https://www.esrf.fr/id29) and will be followed by MicroMAX at MAX IV (https://www.maxiv.lu.se/micromax).

Photoreactions of proteins do not necessarily proceed in the crystalline state exactly as in solution (see, for instance, the identification of an intermediate state that exists in the *in crystallo* photocycle of PYP but not in the photocycle in solution; Konold *et al.*, 2020 ▸), hence it is highly desirable to perform spectroscopic experiments on crystalline samples prior to any TR-MX experiment. This helps to validate the rise and decay of various intermediates so that relevant time points can be chosen (see for instance, the build-up of the late L and M intermediate states in the photocycle of bacterio­rhodopsin; Nango *et al.*, 2016 ▸). Moreover, it has recently been pointed out that the absorption of multiple photons from the pump laser could affect the photoreaction and potentially lead to artefacts in the interpretation of structural intermediates (Miller *et al.*, 2020 ▸; Barends *et al.*, 2022 ▸). Power-titration spectroscopic experiments prior to the diffraction experiment should help to identify these potential artefacts.

In order to prepare and support TR-SSX experiments, we have built a setup that is capable of measuring time-resolved UV–Vis absorption spectra on macromolecular crystals on the microsecond-to-second timescale. The TR-*ic*OS instrument is located in the *ic*OS Lab at the ESRF (https://www.esrf.fr/icOS; von Stetten *et al.*, 2015 ▸) beside the new serial crystallography beamline ID29-SMX. The *ic*OS Lab groups together a number of instruments that can be used to perform various types of spectroscopies (UV–Vis absorption, fluorescence emission, Raman) on crystals at cryogenic or room temperature, either in a static or slow-time-resolved (time resolution of tens of milliseconds) manner. TR-*ic*OS has been developed to identify spectroscopic transitions occurring *in crystallo* for a given photoactivatable biological system upon nanosecond light activation. The setup has two main purposes in future TR-MX experiments: it will facilitate the identification of interesting delays and it will provide complementary information to cross-validate diffraction data.

## 2. Instrumentation

**Figure 2:**
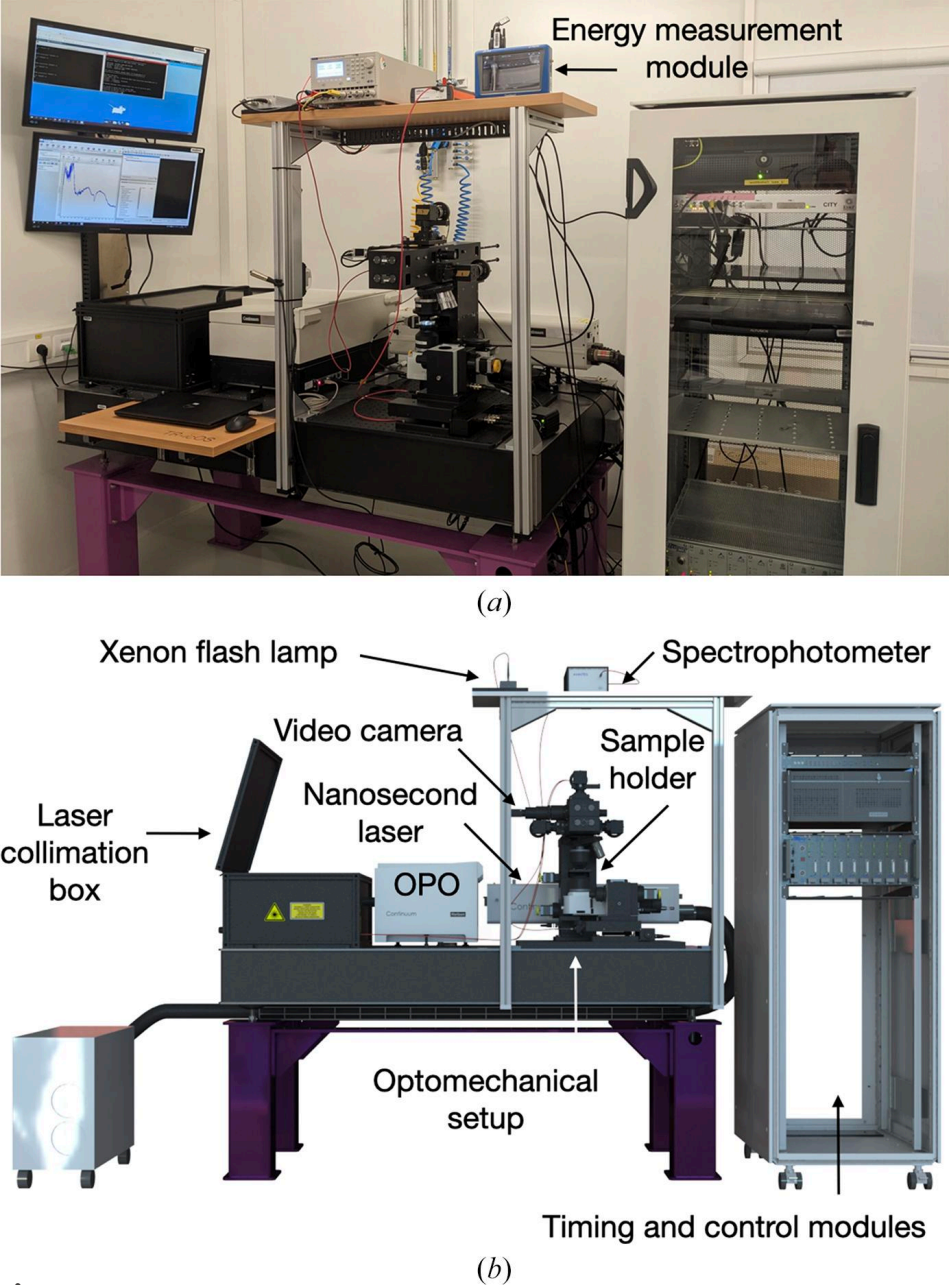
Overview of the TR-*ic*OS setup installed at the *ic*OS Lab at the ESRF. (*a*) Photograph and (*b*) *SOLIDWORKS* rendering of the experimental setup.

The TR-*ic*OS instrument is composed of eight main elements (Figs. 2 ▸ *a* and 2 ▸ *b*): the nanosecond laser used as the pump, the optical box for laser coupling, the xenon flash lamp used as the probe, the optomechanical setup for illumination of the sample, the sample holder, the energy-measurement module, the spectrophotometer and the timing module. All of these are highlighted in Fig. 2 ▸(*b*) and are described in the following paragraphs.

### 2.1. Tuneable nanosecond laser

The nanosecond laser is composed of a control unit (CU) located outside the *ic*OS Lab. The CU includes a 2.5 kW chiller using ethylene glycol solution as a coolant. The 355 nm wavelength Surelite EX laser (Amplitude Technologies, Evry, France) generates pulses of 3–5 ns duration with a maximum energy of 220 mJ per pulse and with a fixed 10 Hz repetition rate. The Horizon II OPO (optical parametric oscillator; Amplitude Technologies, Evry, France) converts the 355 nm laser light into a lower energy laser light that can be tuned between 400 and 2750 nm wavelength. Pulses from the OPO also have a duration of 3–5 ns with a maximal repetition rate of 10 Hz. The maximal pulse energy is wavelength-dependent (80 mJ at 425 nm and 30 mJ at 700 nm).

### 2.2. Xenon flash lamp

**Figure 3:**
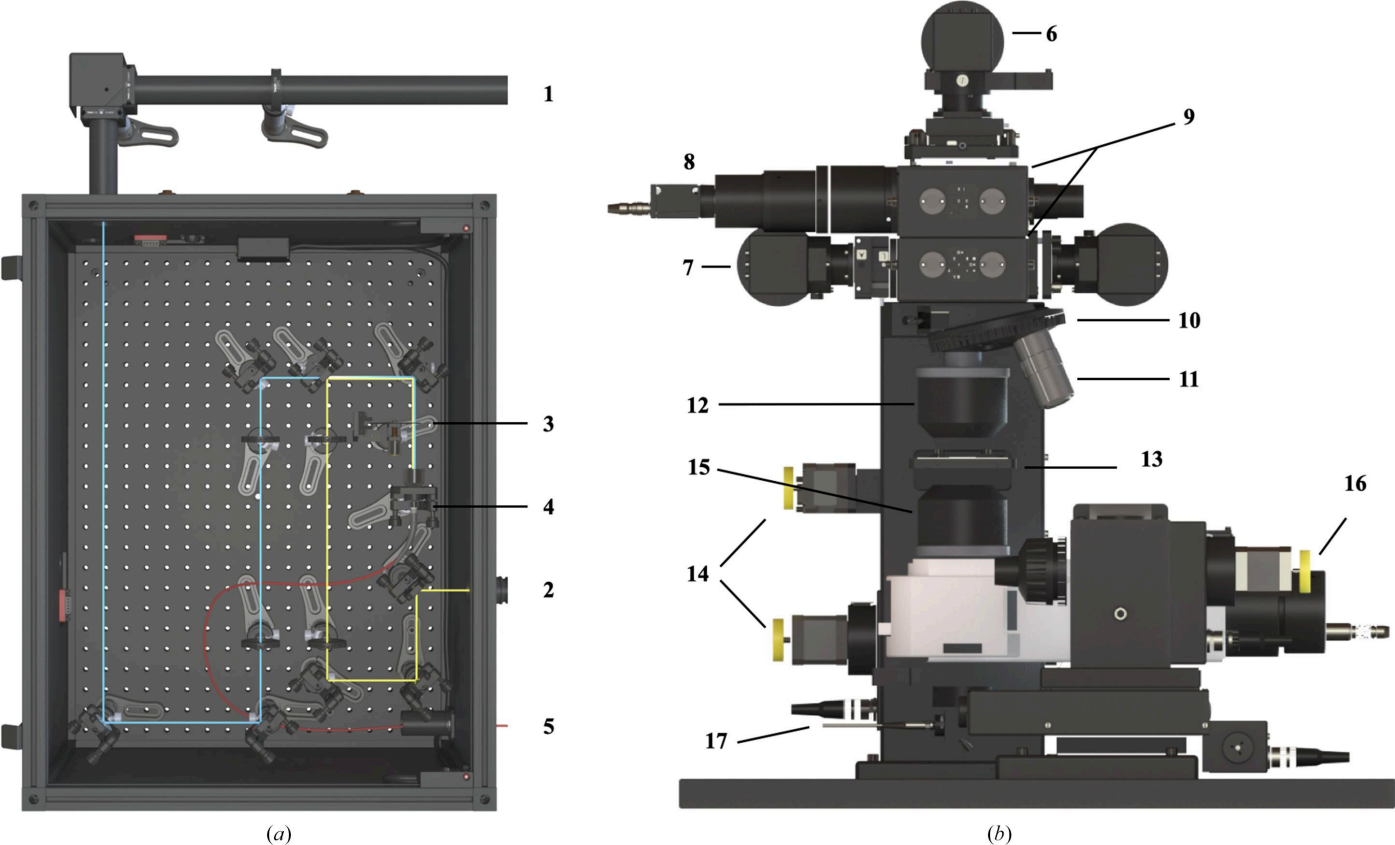
Schematics of (*a*) the optical box, highlighting the UV and Vis–NIR optical paths (blue and yellow, respectively), and (*b*) the optomechanical setup, in which measurements are performed. Details of the various items are as follows. (**1**) Incoming pulses from the (355 nm) output of the OPO. (**2**) Incoming pulses from the (400–2750 nm) output of the OPO. (**3**) Energy-attenuating wheel. (**4**) Collimating lens. (**5**) High-energy optical fibre. (**6**) SMA port for connecting the fibre coming from the optical box. (**7**) SMA port for connecting the fibre coming from the xenon flash lamp. (**8**) Video camera. (**9**) Drawers containing semi-transparent mirrors, polka-dot mirrors and neutral filters. (**10**) Revolver objective mount. (**11**) 2× and 10× microscope objectives. (**12**) Top 15× reflective objective. (**13**) Sample stage. (**14**) Two of the three translation-stage motors of the sample holder. (**15**) Bottom 15× reflective objective. (**16**) One of the three translation-stage motors of the bottom objective. (**17**) SMA port for connecting the fibre to the spectrophotometer.

The 5 W xenon flash-lamp module (L11316-11; Hamamatsu Photonics, Japan) generates white-light pulses peaking at ∼1.3 µs, with a full width at half maximum (FWHM) of ∼1.2 µs. The flash-lamp pulse has a tail extending its effective duration beyond 5 µs; however, most of its energy (∼80%) is emitted before ∼2 µs. The module is connected to the left port of the optomechanical setup (Fig. 3 ▸ *b*) via a 400 µm diameter broadband UV–Vis–NIR optical fibre (Avantes, Apeldoorn, The Netherlands).

### 2.3. Laser-fibre coupling

The function of the so-called ‘optical box’ is primarily to couple the two laser outputs of the OPO box to the entrance of an optical fibre. The corresponding optical paths are highlighted by the blue (355 nm output, ‘UV path’) and yellow (400–2750 nm, ‘Vis–NIR path’) traces in Fig. 3 ▸(*a*). The UV path is composed of a set of BB1-E01 mirrors (Thorlabs, New Jersey, USA). The Vis–NIR path is composed of BB1-E02 mirrors (Thorlabs, New Jersey, USA). For both paths, mirrors are mounted in KM100 mounts and the laser pulses are fed into a high-energy 910 µm diameter optical fibre (model MHP910L02, Thorlabs, New Jersey, USA) via a collimating lens (model COL-UV/VIS-25, Avantes, Apeldoorn, The Netherlands). As shown in Fig. 3 ▸(*a*), space remains available in the optical box for future developments [for example the installation of a CW (continuous wave) laser for steady-state actinic illumination].

### 2.4. Optomechanical setup

The optomechanical setup, in which measurements are performed, is shown in Fig. 3 ▸(*b*). The nanosecond laser and the xenon flash lamp are connected via optical fibres reaching SMA optical fibre ports that are coupled to off-axis parabolic mirrors in order to produce collimated beams that are superimposed by construction (labels **6** and **7** in Fig. 3 ▸ *b*). These beams traverse two drawers (**9**), offering various combinations to distribute the light signal towards a video camera (**8**), a white-light output and the top objective via a series of metallic mirrors, polka-dot and plate beamsplitters. Sample visualization for alignment purposes is achieved with a colour video camera (model ACA1300 30GC, Basler, Ahrensburg, Germany; **8**) using one of the two microscope objectives (**11**) on the revolver mount (**10**). The third objective (15×, infinite-corrected) is of the reflective type (**12**) and is thus suited for spectroscopic measurements. The sample holder (**13**) is mounted on a three-axis motorized adjustable sample stage (**14**). The bottom reflective objective (15×, corrected for an output focal distance of 160 mm) is also mounted on a three-axis motorized support in order to optimize the collection of the light transmitted through the sample. The resulting signal is output at the lower SMA port (**17**), which is connected to the spectrophotometer via an optical fibre (Section 2.7). The optomechanical setup was designed and fabricated by Optique Peter, Lyon, France and is inspired by the Cal(ai)2doscope instrument installed at the Institut de Biologie Structurale, Grenoble, France (Byrdin & Bourgeois, 2016 ▸).

### 2.5. Sample holder

**Figure 4:**
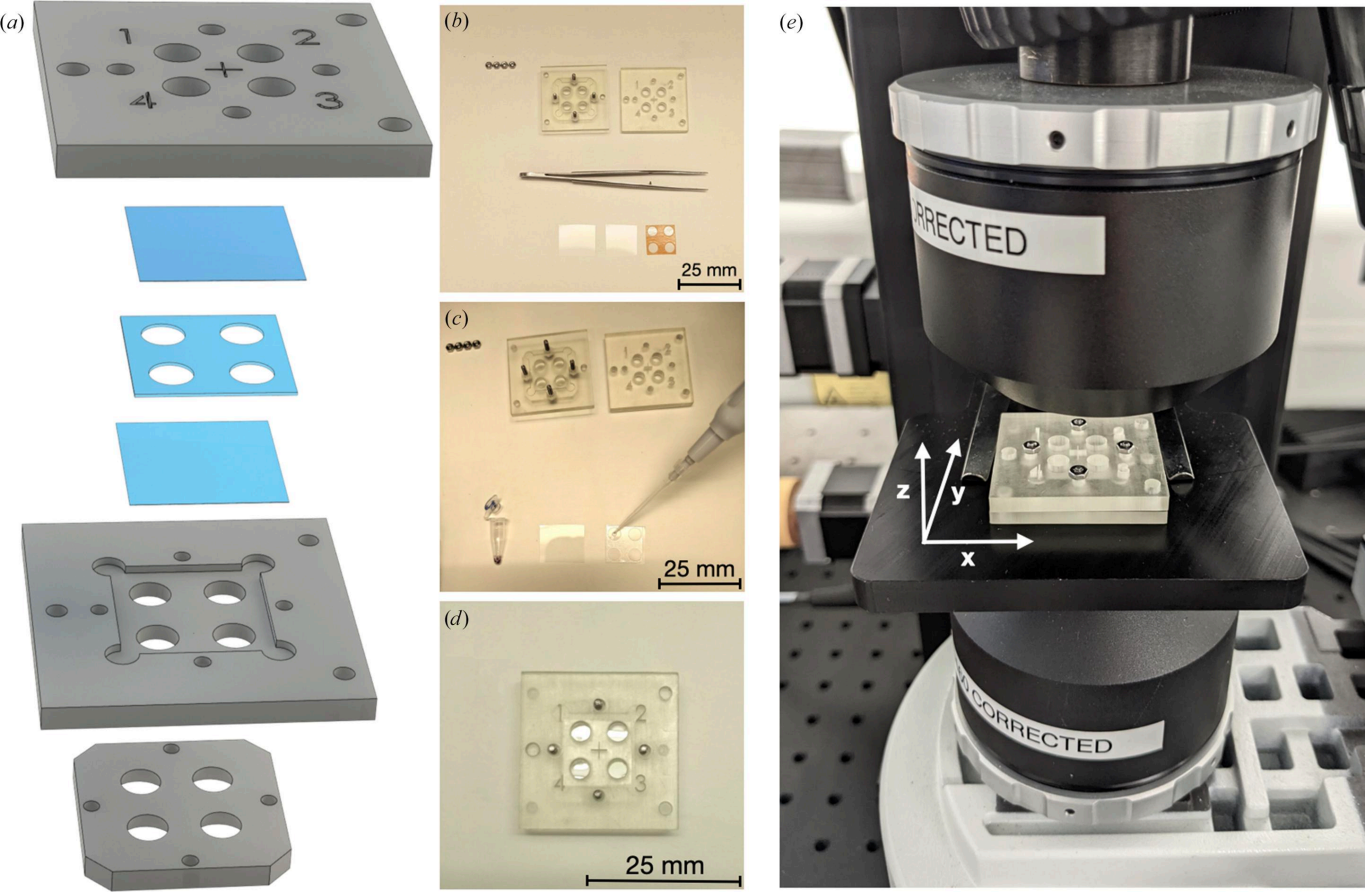
The sample holder for microcrystals. (*a*) Exploded view of the sample holder composed of 3D-printed parts (grey), COC film and perforated double-sided sticky tape (blue). Each sample hole has a diameter of 5 mm. (*b*, *c*, *d*) Snapshots of sample-holder assembly. (*e*) Sample holder mounted on the sample stage.

The sample holder (Fig. 4 ▸) was designed in-house and 3D-printed on an Asiga Pico2 printer using 3Dresyns UHF resin. The crystals are mounted between two layers of 100 µm thick cyclic olefin copolymer (COC) film and 60 µm thick double-sized sticky tape provided by SWISSCI (Buckinghamshire, UK) under references LCP-UV100 and LCP-100, respectively.

### 2.6. Tuning of nanosecond laser-pulse energy at the sample position

**Figure 5:**
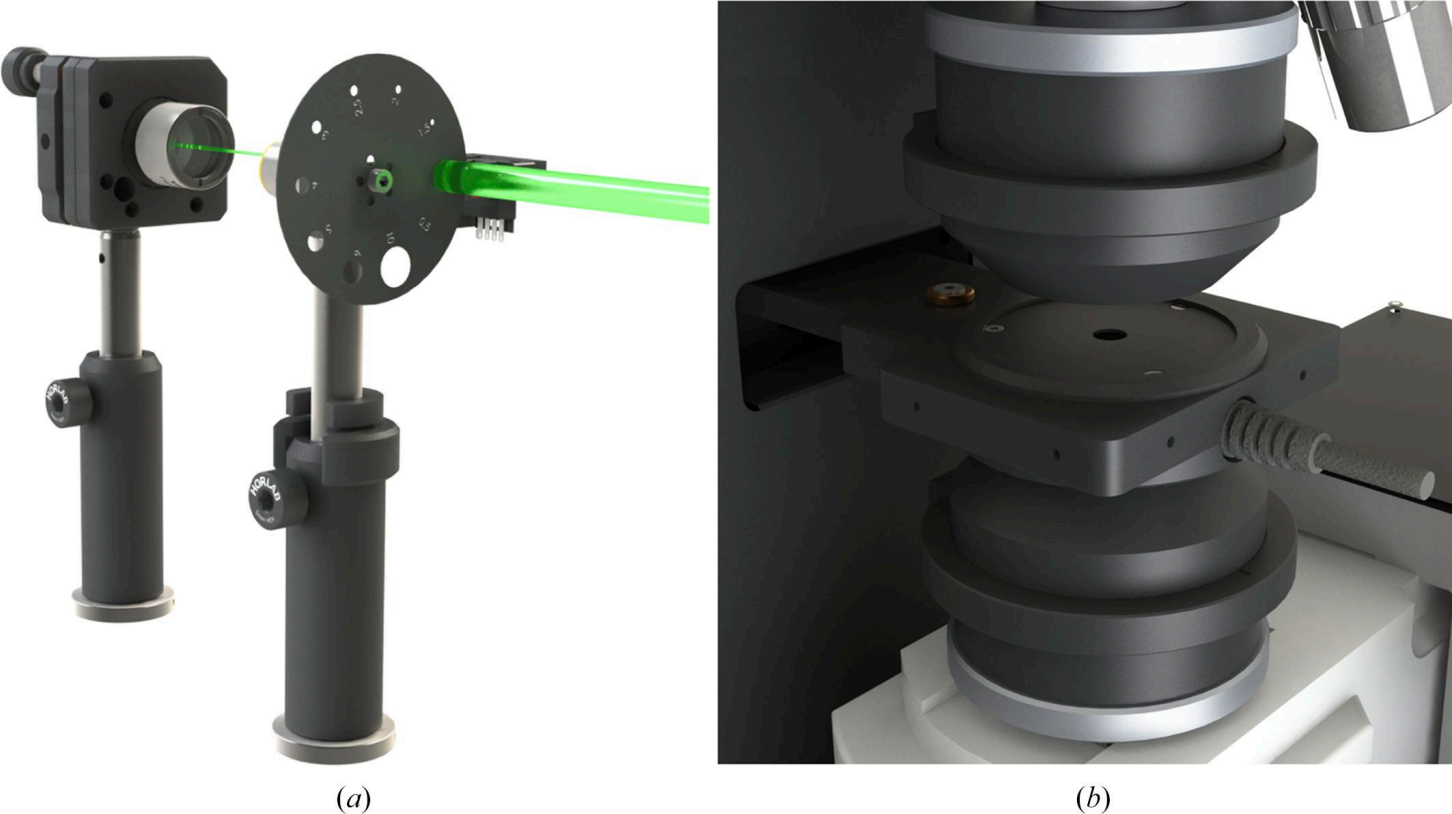
(*a*) Energy-attenuating wheel and (*b*) energy-measurement module at the sample position.

The energy of the nanosecond laser pulses reaching the sample can be tuned using three levels of control. A coarse adjustment of the energy range is achieved at the beginning of an experimental session using the Glan–Taylor prism present in the OPO box. Then, in order to easily modulate the energy of the nanosecond laser pulses reaching the sample, we have designed a motorized energy-attenuating wheel comprising ten apertures with increasing diameters from 0.5 to 10 mm (Fig. 5 ▸ *a*). This wheel (**3**) is located ahead of the collimating lens (**4**) (Fig. 3 ▸ *a*). Moving from the smaller to the wider aperture allows the straightforward coverage of slightly more than one order of magnitude in peak fluence (or energy density), typically from 40 to 1000 mJ cm^−2^. A final fine-tuning around an energy of interest can be achieved by using the 20%, 50% and 80% attenuating plate beamsplitters located in one drawer at the top of the optomechanical setup. One unexpected bonus of the use of the energy-attenuating wheel has been to discard the few laser pulses that arrive slightly off-centre onto the collimating lens, eventually hitting the metal part of the SMA connector of the optical fibre and irreversibly damaging it.

Precise measurement of the laser pulse energy at the sample position was achieved using a pyroelectric sensor (PE9-C, Ophir Photonics, Massachusetts, USA; Fig. 5 ▸ *b*), for which a dedicated holder was designed. For pulse-energy measurements the sensor holder replaces the sample holder and places the sensor surface at the exact height as the putative sample position. This sensor is designed for the measurement of very low energies ranging from 0.2 µJ to 1 mJ. A stand-alone Ophir Centauri control unit allows the recording and averaging of multiple pulses. During energy measurements, a series of ten pulses are systematically averaged.

The diameter of the laser spot at the sample position was determined to be 87.8 ± 4.2 µm (FWHM) or 128.2 ± 4.3 µm (1/*e* ^2^ cutoff) using a beam-profiling camera (WinCamD-LCM, DataRay, Redding, California, USA). These values do not vary with pinhole size. Likewise, the diameter of the xenon flash-lamp spot at the sample position was determined to be 44.3 ± 1.0 µm (FWHM) or 65.1 ± 2.7 µm (1/*e* ^2^ cutoff). The ratio between the two spot diameters is 68.5% and 68.0%, respectively, suggesting a small deviation from a perfect Gaussian profile (58.9%). However, since the slightly defocused beam was shown to exhibit a perfectly Gaussian profile, the small profile deviation at the focal position appears to originate from the convolution of the strongly divergent light beam within the thickness of the sensor.

### 2.7. Spectrophotometer

Spectra are recorded using a spectrophotometer equipped with a CMOS detector (AvaSpec-ULS2048CL-EVO-RS-UA, Avantes, Apeldoorn, The Netherlands), allowing integration times as short as 9 µs. The recording of spectra is triggered by a TTL signal emitted by a CITY timing module.

### 2.8. Timing module

The ESRF-developed CITY timing module (https://bliss.gitlab-pages.esrf.fr/bliss/master/config_city.html) receives the radio-frequency master clock of the ESRF accelerator timing system and provides up to 12 output channels providing single pulses and/or periodic signals whose period, phase and width can be adjusted and synchronized with the bunch structure of the ESRF–EBS ring. The reason for having such a system on TR-*ic*OS is that the same timing module is used on the ID29-SMX beamline, thus facilitating the transfer of configurations between spectroscopic and diffraction experiments. The CITY module is packaged as a standalone 19-inch unit mounted in the dedicated TR-*ic*OS electronics rack (Fig. 2 ▸ *b*). It provides the master clock of the instrument that controls four different TTL signals (0–5 V, 10 µs width; see Section 3.2).

## 3. Control software

**Figure 6:**
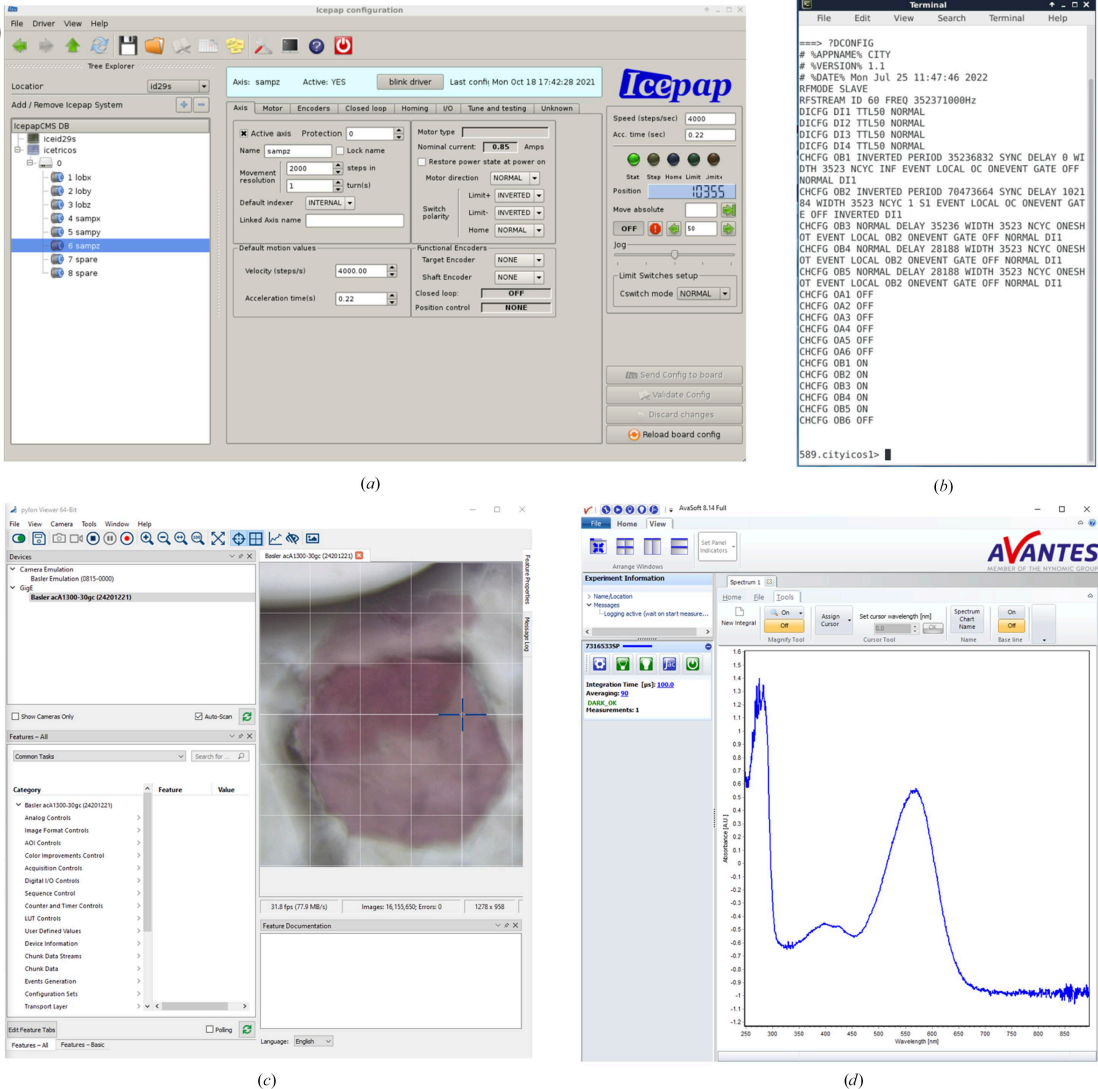
Software windows for (*a*) motor control, (*b*) experiment timing, (*c*) sample visualization (a square represents a 50 × 50 µm^2^ area) and (*d*) spectroscopic data acquisition.

The control of motor positions is performed via the *IcePAP* graphical interface (https://www.esrf.fr/Instrumentation/DetectorsAndElectronics/icepap; Fig. 6 ▸ *a*). The event sequence is provided to the CITY timing module via a terminal (Fig. 6 ▸ *b*). Samples are visualized through the *PYLON* software controlling the Basler camera (Fig. 6 ▸ *c*). Spectra are recorded using the *AvaSoft* software (Avantes; Fig. 6 ▸ *d*).

### 3.1. Motor control

The position of the sample stage (*X*, *Y*, *Z*) and the position of the bottom reflective objective (*X*, *Y*, *Z*) are controlled by stepper motors associated with high-precision scanning and positioning stages allowing both submicrometric positioning of the elements and repeatability of displacements. Translations of the sample stage along the *X* and *Y* axes are ensured by high-precision linear stages (model LIMES 60-20-HMS, Owis, Staufen im Breisgau, Germany). Translation of both the sample stage and the bottom objective along the *Z* axis is ensured by motorized microscope modules (model BXFM, Olympus, Tokyo, Japan). Translations of the bottom objective along the *X* and *Y* axes are ensured by high-precision linear stages (model MT 50 × 50, Märzhäuser, Wetzlar, Germany) motorized with stepper motors (model Vexta PK245-01B, Oriental Motor, Tokyo, Japan). The rotation of the energy-attenuating wheel is ensured by a stepper motor (model 1321, Faulhaber, Schönaich, Germany) and the repeatability of the rotation is ensured by a homing system consisting of a through-beam photoelectric sensor (model EE-SX671OMC, Omron, Tokyo, Japan) detecting a notch in the external rim of the wheel.

### 3.2. Timing of the experimental events

**Figure 7:**
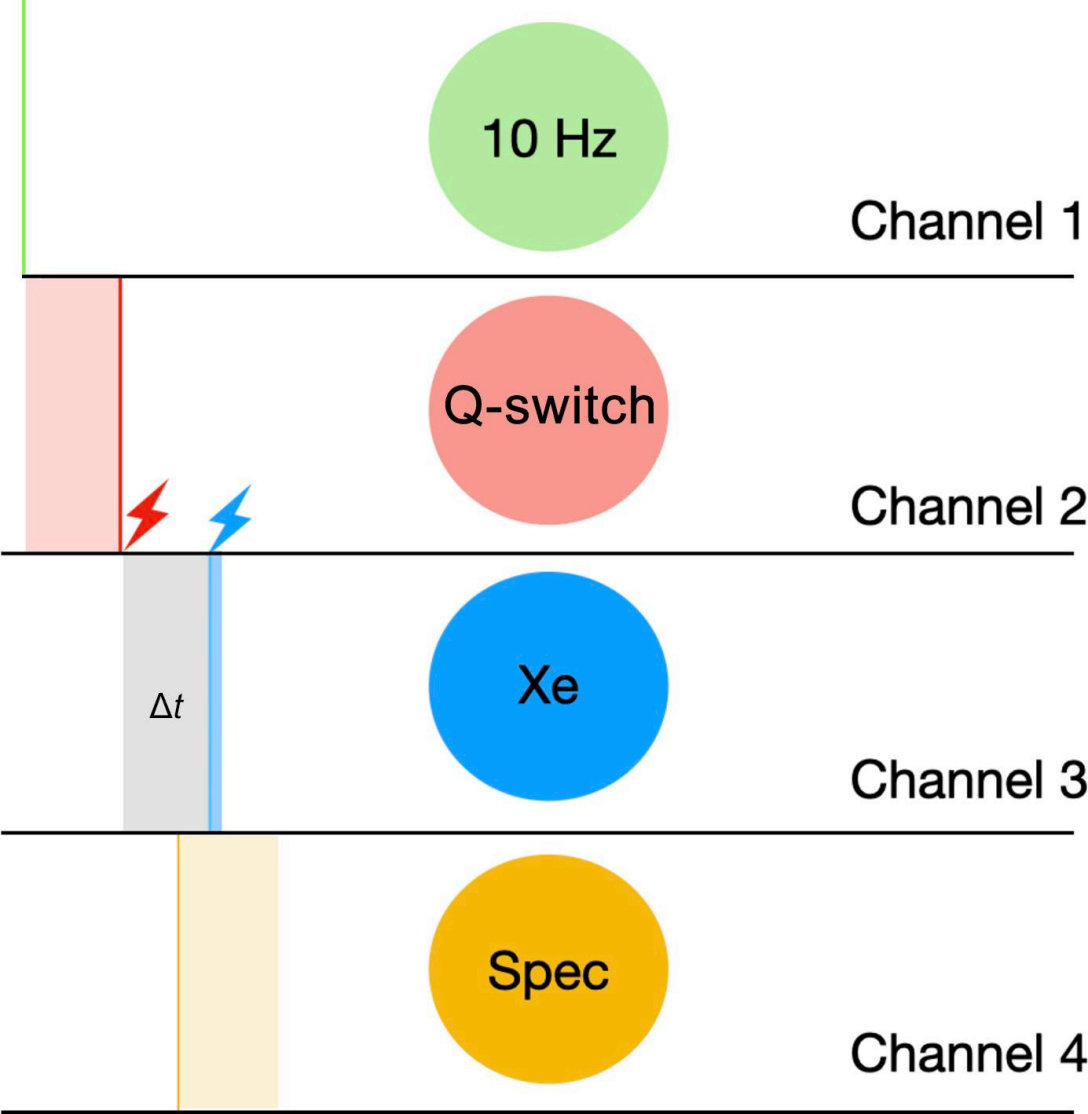
Timing of the events controlled by the CITY module. The Q-switch delay is typically 290 µs (light red), the laser-pulse duration is 3–5 ns (dark red), the pump–probe time delay is usually varied between 2 µs and 12 s (grey), the xenon-flash lamp pulse duration is 2 µs (blue) and the spectrophotometer acquisition window is set to 100 µs (orange).

The CITY timing module delivers sequences of TTL signals via four outgoing channels (Fig. 7 ▸). Two of these signals control the nanosecond laser, first by providing a constant 10 Hz to pump the Yb:YAG rod (channel 1) and then by triggering the Q-switch (channel 2). The Q-switch is a technique used to generate high-energy, short-duration pulses that is based on a variable attenuator placed inside the optical cavity of a laser. After population inversion has been reached, stimulated light emission cannot occur until there is rapid change from a low-quality (low-Q) to a high-quality (high-Q) gain medium. Channel 3 triggers the xenon flash-lamp module. In practice, the delay between events (2) and (3) corresponds to the time delay of the pump–probe experiment. Channel (4) activates spectroscopic recording by the spectrophotometer. It is systematically activated a few microseconds before the xenon flash lamp to prevent any possibility of detector jitter: 2 µs for delays lower than 100 µs and 20 µs for delays longer than 100 µs (to exclude laser scattering on the longer time points). When ‘ground-state’ spectra are needed (that is, without actinic excitation), a shutter is manually closed in the OPO box to block the pump signal.

### 3.3. Spectroscopic data acquisition and analysis

Spectroscopic data are recorded using the *AvaSoft* software (version 8.11; Avantes). Data acquisition is performed using the triggering mode of the spectrophotometer. By default, a 100 µs acquisition time is used to record the ‘dark’ spectrum (the background signal of the CMOS detector), the ‘reference’ spectrum (the light transmitted next to a crystal) and the ‘sample’ spectrum (the light transmitted through a crystal). A UV–Vis absorption spectrum is straightforwardly calculated using these three spectra. In-house Python scripts were written to average, smooth and/or plot (https://github.com/ncara/TRicOS; Caramello *et al.*, in preparation).

## 4. Experimental procedure

Two types of samples can be loaded: either single crystals obtained in a crystallization plate or slurries of microcrystals obtained in batches. In the former case, 2 µl of the crystallization mother liquor is loaded into one well of the sample holder and a handful of crystals are manually transferred into this drop using a crystallization loop. In the latter case, the microcrystal slurries are diluted several times and 2 µl of each resulting solution is loaded into a different well of the sample holder. The microscope objectives are first used to locate a suitable crystal upon iterative horizontal translation of the sample stage. The sample *Z* position is then adjusted by vertically translating the stage until a sharp image is obtained with the camera using the reflective objective, meaning that both the laser and flash-lamp focal spots are positioned at the top surface of the crystal. However, having inserted a large object between the two reflective objectives with a refractive index different from that of air, the focal volumes (of ∼20 µm thickness) of both objectives no longer match. Thus, the bottom objective is then translated along the *Z* axis until the transmitted light signal is maximized. From then onwards, only minimal recentring is needed to move from one crystal to the other within a given well of the sample holder. Reference and dark spectra are first recorded in the vicinity of the crystal of interest and are used for all subsequent UV–Vis absorption spectra. A typical pump–probe experiment starts with the recording of ground-state spectra. The OPO shutter is then manually opened and a series of pump–probe spectra can be obtained by varying the delay between the nanosecond laser and xenon flash-lamp pulses in the CITY module terminal. The resulting pump–probe spectra series corresponds to a given laser pulse energy and can be repeated at different energies by rotating the energy-attenuating wheel.

## 5. Results

We used the membrane proton pump bacteriorhodopsin from *Halobacterium salinarum* (BR) as a benchmark photoactive protein for our setup. The photocycle of BR in its native environment, the so-called purple membrane, develops over many orders of magnitude in time from the spectroscopic intermediate states I (rise time of ∼200 fs), J (∼450 fs), K (∼4 ps), L (∼1 µs), M_1_ (∼40 µs), M_2_ (∼350 µs), N (∼5 ms) and, finally, O (∼5 ms), as determined by various spectroscopic methods (Doig *et al.*, 1991 ▸; Neutze *et al.*, 2002 ▸). The whole photocycle is completed within ∼20 ms and is repeatable. However, the rise times of these intermediate states vary between the purple membrane and a crystalline environment (Efremov *et al.*, 2006 ▸; Weinert *et al.*, 2019 ▸); thus, UV–Vis absorption spectroscopy experiments directly performed on crystals are required in order to determine the time points of interest in TR-MX experiments on BR crystals. The time resolution of our instrument (2 µs) is suited to probe the build-up of late intermediates, starting with the M_1_ state.

**Figure 8:**
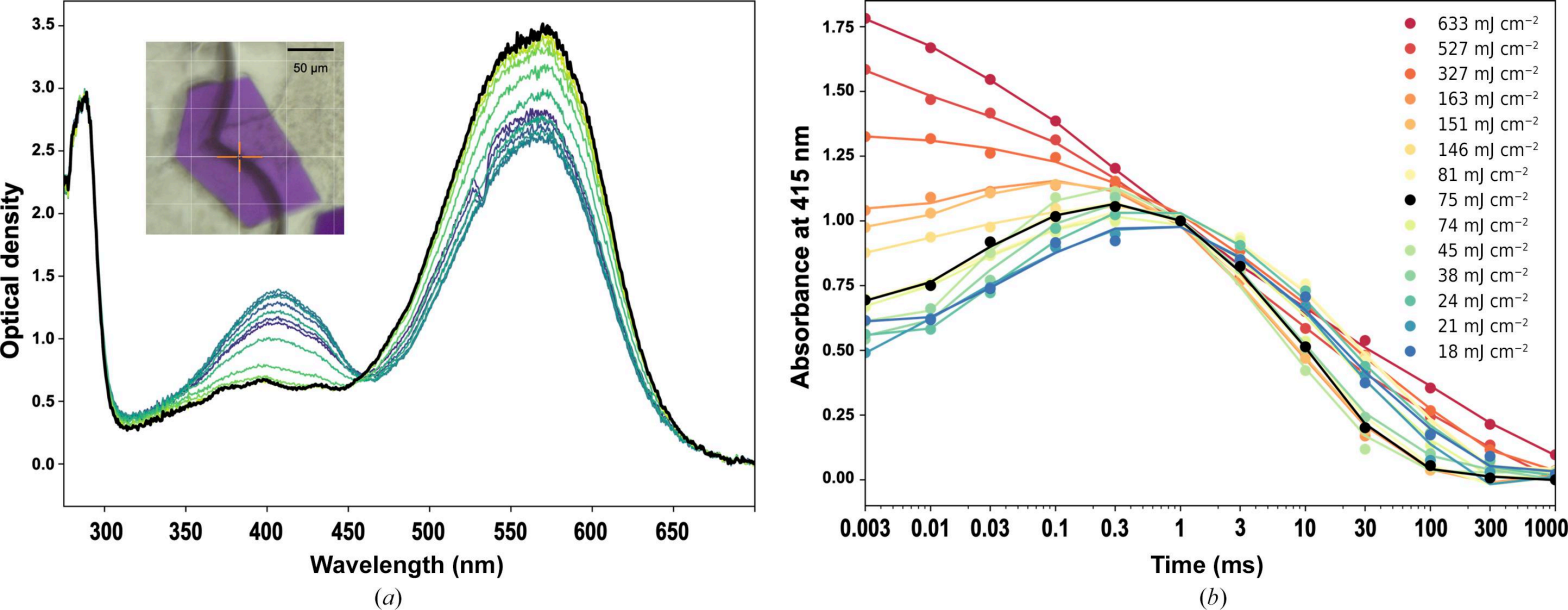
(*a*) Time-resolved series of UV–Vis absorption spectra obtained on a single BR crystal (see insert) with delays varying logarithmically between 3 µs and 1 s (purple to blue to green). The initial ground-state spectrum is shown as a thick black line. The excitation wavelength is 532 nm and the pulse energy is 75 mJ cm^−2^ at the sample position. (*b*) Power titration of the M-state time evolution. For each spectrum of the time series recorded at a given fluence, the optical density at 415 nm (OD_415_, from which the OD_415_ of the ground state has been subtracted) is plotted as a function of time. The resulting profiles have been scaled to each other using the OD_415_ value at 1 ms. The black trace corresponds to the profile of the experiment depicted in (*a*).

Crystals of BR, expressed and purified as described by Gordeliy *et al.* (2003 ▸), were crystallized as described by Borshchevskiy *et al.* (2022 ▸), resulting in hexagonal plates with dimensions of ∼50–200 × 50–200 × 10–20 µm^3^ (Fig. 8 ▸ *a*). Crystals were placed in the sample holder after manual harvesting in the lipidic cubic phase (LCP) using a standard MiTeGen loop (https://www.mitegen.com).

In order to identify the time range during which occupancy of the M state is maximal, we recorded UV–Vis absorption spectra with pump–probe delays ranging from 3 µs to 1 s (Fig. 8 ▸ *a*). A given experiment is performed at a certain pulse energy (75 mJ cm^−2^; Fig. 8 ▸ *a*). To assess complete reversibility of the photoreaction and thus the possibility of performing several time-resolved measurements on the same crystal, a ground-state spectrum is recorded prior to a pump–probe transient spectrum. The lack of bleaching of the photoactive protein can be verified by the iterative superimposition of successive ground-state spectra. The build-up and decay of the M state can be visualized by monitoring the absorbance at 415 nm. As expected, the M state is already present in the crystal 3 µs after light excitation, and its occupancy is maximal between 100 µs and 1 ms (black trace in Fig. 8 ▸ *b*). The build-up/decay profile matches published data recorded on BR crystals (Efremov *et al.*, 2006 ▸).

This experiment was repeated at various laser-pulse energies, thus producing a power titration (or, more rigorously, a peak-fluence titration) of the M-state build-up and decay profile. The peak fluence was varied from 18 to 633 mJ cm^−2^ (calculations were performed as in Nass Kovacs *et al.*, 2019 ▸). The resulting profiles can be divided into three groups. The first, grouping seven experiments with fluences between 18 and 75 mJ cm^−2^, resembles the profile determined by Efremov *et al.* (2006 ▸), except that the ratio of the respective M-state amplitudes at 10 µs and 300 µs (close to the maximum M-state occupancy) is ∼60% in the former and ∼30% in the latter. The second groups three experiments with fluences between 81 and 151 mJ cm^−2^, for which the profile is distorted with a 10 µs:300 µs amplitude ratio nearing 100%. Finally, the third group (between 163 and 633 mJ cm^−2^) exhibits a profile that clearly deviates from the expected profile, with a monotonous decay from a maximum occupancy at 3 µs.

These results suggest that the fluence used for the first profile group corresponds to a functional photocycle in the crystals and that increasing fluences lead to the build-up of an artefactual M state, which, we postulate, could well correspond to direct chromophore deprotonation upon multiphoton absorption. The population of this artefactual M state adds up to that of the expected M state at early time points (the second group) and then eventually dominates (the third group). This suggests limiting peak fluences in diffraction experiments to values below 100 mJ cm^−2^. Fortunately, a number of recent TR-MX experiments performed on BR at XFELs and synchrotrons have been conducted using fluences in this regime. Ultrafast intermediate states I to K have been probed at 42 mJ cm^−2^ (Nogly *et al.*, 2018 ▸) and 69 mJ cm^−2^ (Nass Kovacs *et al.*, 2019 ▸) (these values were calculated without taking into account scattering from the carrying medium). Later intermediate states K to M have been studied at 110 mJ cm^−2^ (Nango *et al.*, 2016 ▸). Finally, the large structural changes of the late intermediate states M_2_ to N have been visualized at 50 mJ cm^−2^ (recalculated from Weinert *et al.*, 2019 ▸). Of note, the early study focused on *in crystallo* spectroscopic characterization (Efremov *et al.*, 2006 ▸) reported a peak fluence of 3 mJ cm^−2^ and thus constitutes the *in crystallo* study of the BR photocycle with the lowest reported fluence. While the fluence of the Nango study may appear to be slightly higher than the identified threshold, the loss of photons via scattering at the surface, and through the LCP microjet, likely led to an effective fluence below this threshold. Overall, these results confirm the proposition by Brändén and Neutze that multiphoton regimes may not systematically deviate from the single-photon regime and that they could be used to maximize crystallographic occupancy (Brändén & Neutze, 2021 ▸).

## 6. Perspectives

Fourth-generation synchrotrons have reached unprecedented brilliance thanks to their low emittance, offering the possibility of intense monochromatic microsecond X-ray pulses. This, coupled with the recent revival of TR-MX at synchrotrons, has triggered the design of dedicated beamlines, starting with ID29 at the ESRF and MicroMAX at MAX IV, at which microsecond time resolution can be achieved. Other existing state-of-the-art beamlines already performing millisecond time-resolution TR-MX, such as TREXX at PETRA III (Schulz *et al.*, 2018 ▸), I24 at Diamond Light Source (Baxter *et al.*, 2022 ▸) and X06SA at the Swiss Light Source (Weinert *et al.*, 2019 ▸), will likely be upgraded so they can also achieve microsecond time resolution. This should bolster the field of dynamic photobiology as studied by TR-MX. In order to best prepare diffraction experiments on photoactive proteins, prior characterization of crystals by time-resolved spectroscopy is essential for the identification of time points of interest and for optimization of the choice of laser fluence. In particular, the latter should help to maximize intermediate-state occupancy in the diffraction data, while preventing or minimizing the possibility of artefactual photoreactions. We have built the TR-*ic*OS instrument to serve these purposes. It is also meant to evolve, for instance to probe flowing microcrystals in a suitable microfluidics device and, using photocaged substrates or cofactors, to exploit non-photoactive proteins in which the UV–Vis absorption properties of a certain chemical group evolve with time.
